# Intracellular calcium signals display an avalanche-like behavior over multiple lengthscales

**DOI:** 10.3389/fphys.2012.00350

**Published:** 2012-09-03

**Authors:** Lucía Lopez, Estefanía Piegari, Lorena Sigaut, Silvina Ponce Dawson

**Affiliations:** Departamento de Física FCEN-UBA and IFIBA, Ciudad Universitaria, Pabellón IBuenos Aires, Argentina

**Keywords:** calcium signals, puffs, phase transition, self-organized criticality, percolation

## Abstract

Many natural phenomena display “self-organized criticality” (SOC), (Bak et al., 1987). This refers to spatially extended systems for which patterns of activity characterized by different lengthscales can occur with a probability density that follows a power law with pattern size. Differently from power laws at phase transitions, systems displaying SOC do not need the tuning of an external parameter. Here we analyze intracellular calcium (Ca^2+^) signals, a key component of the signaling toolkit of almost any cell type. Ca^2+^ signals can either be spatially restricted (local) or propagate throughout the cell (global). Different models have suggested that the transition from local to global signals is similar to that of directed percolation. Directed percolation has been associated, in turn, to the appearance of SOC. In this paper we discuss these issues within the framework of simple models of Ca^2+^ signal propagation. We also analyze the size distribution of local signals (“puffs”) observed in immature *Xenopus Laevis* oocytes. The puff amplitude distribution obtained from observed local signals is not Gaussian with a noticeable fraction of large size events. The experimental distribution of puff areas in the spatio-temporal record of the image has a long tail that is approximately log-normal. The distribution can also be fitted with a power law relationship albeit with a smaller goodness of fit. The power law behavior is encountered within a simple model that includes some coupling among individual signals for a wide range of parameter values. An analysis of the model shows that a global elevation of the Ca^2+^ concentration plays a major role in determining whether the puff size distribution is long-tailed or not. This suggests that Ca^2+^-clearing from the cytosol is key to determine whether IP_3_-mediated Ca^2+^ signals can display a SOC-like behavior or not.

## 1. Introduction

Ca^2+^ signals are involved in a large variety of physiological processes (Berridge et al., 1998). The diversity of spatio-temporal behaviors that the Ca^2+^ concentration can display is fundamental for the universality of the signals (Berridge and Dupont, 1994; Berridge et al., 2000). The prolonged permanence of high free Ca^2+^ concentrations in the cytosol leads to cell death. For this reason, the signals are built upon local Ca^2+^ elevations due to Ca^2+^ entry from the extracellular medium or internal stores through specialized channels. Depending on different factors, these local elevations can remain local or induce the opening of other Ca^2+^ channels giving rise to signals that propagate over larger distances (Bootman et al., 1997; Callamaras et al., 1998; Sun et al., 1998). Ca^2+^ release from the endoplasmic reticulum (ER) through inositol 1,4,5-trisphosphate (IP_3_) receptors (IP_3_R's) is a key component that participates of both local (e.g., “puffs”) and global (waves) Ca^2+^ signals (Berridge, 1993; Choe and Ehrlich, 2006). There is an extensive literature on the observation of this type of signals which have led to the picture that IP_3_R's are organized in clusters on the membrane of the ER (Smith and Parker, 2009). Although the spatial distribution of IP_3_R's is currently under debate (see e.g., Taufiq-Ur-Rahman et al., 2009), it is apparent that those that are activatable (i.e., can become open) are indeed distributed inhomogeneously (Smith et al., 2009). Therefore, Ca^2+^ signals are characterized by very diverse lengthscales: the typical size of the channel pore (~1 nm); the dimensions of the IP_3_R cluster (~100 nm); the extent of the cell (10 μm−1 nm). Whether the signal remains local or propagates throughout the cell depends on Ca^2+^ itself, since IP_3_R's become open upon Ca^2+^ and IP_3_R binding. Ca^2+^, in turn, plays a dual role, inducing channel opening at moderate concentrations and channel closing at higher ones (Foskett et al., 2007). When a channel becomes open, Ca^2+^ rapidly flows in and diffuses inside the cytosol. Diffusion is not free given that the cells have several mechanisms (among them, binding to immobile or slowly moving “buffers”) to reduce Ca^2+^ concentration. In any case, the local elevation of [Ca^2+^] that originates at an open channel eventually spreads out in space inducing new channel openings if it finds IP_3_-bound IP_3_R's on its way. This Calcium-Induced-Calcium-Release (CICR) is basic for Ca^2+^ signal propagation.

The fact that IP_3_R-clusters are not evenly distributed inside cells introduce a new factor that also modulates the ability of a signal to propagate or not: the mean inter-cluster distance, *d*. In Keizer et al. (1998); Pearson and Ponce-Dawson (1998); Ponce Dawson et al. (1999), we introduced the simple deterministic “fire-diffuse-fire” (fdf) model to analyze the effect of this additional variable. In particular we found different wave front regimes depending on the ratio between the typical time a cluster remains active (releasing Ca^2+^) and the time it takes for Ca^2+^ to diffuse over the distance *d*. The existence of an inter-cluster separation can also lead to propagation failure even if some clusters are able to release Ca^2+^. In Pearson and Ponce-Dawson (1998), we analyzed propagation failure within the framework of the deterministic fdf model. We found that the dynamics of the wave became irregular with its temporal pattern undergoing a series of period-doubling bifurcations until it eventually disappeared as an attracting solution of the model. Propagation failure was also studied within the framework of stochastic fdf models (Coombes and Timofeeva, 2003; Calabrese et al., 2010). These stochastic models reproduce better the experimental observations in which, after a series of localized signals, there is a burst of activity that propagates over a much larger spatial region. The relevance of the stochastic localized activity to elicit a global signal has been recently pointed out. In particular, it has been argued that apparently periodic global signals are not truly periodic but are the consequence of stochastic channel openings (Skupin et al., 2008, 2010; Thurley et al., 2011). In fact, it has long been reported that the occurrence of several local signals can eventually elicit a Ca^2+^ wave (Marchant and Parker, 2001). This transition from local to global Ca^2+^ signal propagation ressembles that of percolation (Stauffer and Aharony, 1992), a concept that, although originally referred to fluid displacement in a disordered medium, is now used to describe the behavior of connected clusters of any sort. In the case of the Ca^2+^ signals that we are focusing on in this paper, the latter would correspond to (active) IP_3_R clusters. In systems where the dynamics involves some type of propagation that could evnetually fail, as in the case of Ca^2+^ signals, the relevant concept is that of *directed percolation*. In particular, it has been observed that the stochastic fdf model of (Coombes and Timofeeva, 2003) is in the universality class of directed percolation (Timofeeva and Coombes, 2004). This means that the correlation lengthscale and other properties of the system scale as |*p* − *p*_*o*_|^α^ with specific values of α for *p* ≈ *p*_*o*_, where *p* is a parameter that controls the transition from a macroscopically active (percolating) to an inactive (non-percolating) state (that occur, e.g., for *p* > *p*_*o*_ and *p* < *p*_*o*_, respectively). The exponent, α, depends on the quantity whose dependence with *p* is being analyzed. The values that these exponents take on depend on the type of transition that takes place at *p* = *p*_*o*_ independently of the specificities of the system under study (i.e., they are “universal”). The inhomogenous spatial distribution of IP_3_R's also shapes the kinetics of local signals such as puffs (Diambra and Marchant, 2011). In fact, we investigated the “percolation” of Ca^2+^ signals inside an IP_3_R cluster rather than between clusters in (Solovey and Ponce Dawson, 2010). In that paper we called percolation the transition between a situation for which most signals involved the opening of all IP_3_R's with IP_3_ of the cluster (percolating states) to a situation for which very few IP_3_R's of the cluster opened (non-percolating states). To that end we introduced a very simple model with which we were able to reproduce the experimentally observed puff size distribution reported in Smith and Parker (2009). In particular, by fitting the model to the observations, we determined that, for one open IP_3_R, the radius around which CICR is effective is of the order of the cluster radius (~250 nm). This means a very strong inter-channel coupling due to CICR inside the cluster, but not strong enough so that all IP_3_R's with IP_3_-bound would become open during each puff. Actually, the experimental data seemed to be near the transition between the percolating (IP_3_-dominated) and non-percolating (for which Ca^2+^ is limiting) states. In this paper we investigate this aspect further by analyzing both new experimental data and a simple model that describes inter-cluster coupling. Again in this case the experiments seem to be close to a transition point.

One could argue that finding a natural system at a transition point is non-generic. However, the existence of systems that behave as if they are naturally near a critical point has been widely observed (Bak, 1996). This is the main idea behind “Self-Organized Criticality” (SOC) (Bak et al., 1987, 1988), a property of spatially extended dynamical systems that have a critical point as an attractor. In systems displaying SOC, the state is usually identified to be critical when the lengthscale distribution is scale invariant or follows a power law. The paradigmatic example of a system that displays SOC is the sand pile in which avalanches of all sizes can occur as sand grains are added slowly compared to the typical time of energy dissipation. The existence of avalanches of all possible sizes is similar to the occurrence of Ca^2+^ signals that propagate over different regions and eventually fail to advance. In fact, noise-induced spiral Ca^2+^ waves in astrocytes (Jung et al., 1998) and arrhythmogenic Ca^2+^ waves in cardiac myocytes (Ko et al., 2012) have been shown to display features of SOC. SOC has been argued to occur in several other biological systems (Bak and Sneppen, 1993; Fraiman et al., 2009; Tetzlaff et al., 2010). Directed percolation and SOC have been associated in the literature (Jovanović et al., 1994). For example, a modified version of a model that displays SOC was shown to evolve to a directed percolation critical point (Sornette and Dornic, 1996). In particular, a SOC-like behavior (characterized by a scale invariant distribution) can be reached in systems with a phase transition onto an absorbing (inactive) state that are driven slowly by infinitesimal additions of the quantity that brings the system close to the transition and are allowed to relax upon activation by decreasing this quantity (Dickman et al., 2000; Vespignani et al., 2000). Although this “SOC recipe” is still debatable Pruessner and Peters (2006) it could be the mechanism that underlies the scale-invariant behaviors observed in Ca^2+^ signals as we discuss in this paper.

## 2. Materials and methods

### 2.1. Oocyte preparation

Experiments were performed on immature oocytes from *Xenopus laevis* previously treated with collagenase in order to defolliculate them. Oocytes were loaded by intracellular microinjection 1 h before recording with Fluo-4 (fluorescent calcium indicator), caged InsP_3_ (D-Myo-Inositol 1,4,5-Triphosphate, P4(5)-(1-(2-Nitrophenyl)ethyl) Ester) and the exogenous calcium buffer EGTA (Ethylene glycol-bis(2-aminoethylether)-N,N,N′,N′-tetraacetic acid). Final intracellular concentrations of Fluo-4 and InsP_3_ were 36 μM and 9 μM respectively, assuming 1 μl cytosolic volume. The final concentration of EGTA was varied from 0 μl to 90 μM. Recordings were made at room temperature. All recordings were obtained with the scan line focused at the depth of the pigment granules in the animal hemisphere of the oocyte. Fluo-4 and InsP_3_ were from Molecular Probes Inc.; EGTA was from Sigma Aldrich.

### 2.2. Confocal microscopy

Confocal linescan imaging was performed using a spectral confocal scanning microscope Olympus FluoView1000 that has a spectral scan unit connected to an inverted microscope IX81. Flash photolysis of the caged compound was made with a mercury lamp that comes with the microscope using the modification introduced in Barella et al., 2011. The ultraviolet illumination from the lamp (350–400 nm) enters through an optical fiber that is coupled to a non-conventional port of the microscope in order to perform the photolysis while simultaneously acquiring confocal fluorescence images. The fluorescent indicator was excited using the 488 nm line of a multiline Argon laser that was focused on the oocyte with a ×60 oil immersion objective (NA 1.35). The emitted fluoresecence was detected in the 500–600 nm range. The UV flash was delivered approximately 2 s after having started the image acquisition.

### 2.3. Image analysis

**Figure 1:**
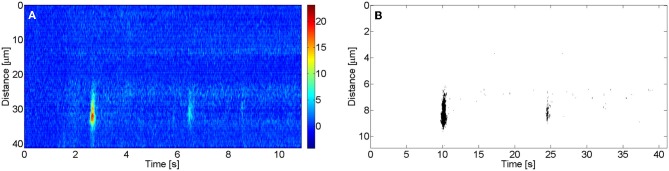
**Linescan image with two puffs in the same site. (A)** Δ*F*/*F*_0_ fluorescence image with the corresponding color code. **(B)** Δ *F*/*F*_0_ thresholded binary image.

All images were analyzed using routines written in MATLAB. Images were smoothed out with a filter prior to this analysis. To determine the amplitude, *A*, of a puff, the mean basal fluorescence at each spatial point, *F*_*0*_, was computed before delivery of the UV flash. The region occupied by a puff was identified manually. The puff amplitude, *A*, was then defined as the maximum value of Δ*F*/*F*_*0*_ (*F* − *F*_0_/*F*_*0*_) in the region, with *F* the fluorescence value at each point. We show in Figure 1A an example of an experimental linescan image with time and space in the horizontal and vertical axes, respectively, and using a color code to display Δ*F*/*F*_*0*_ that goes from colder to warmer colors with increasing fluorescence. In this example two puffs appear at the same release site but at different times, the first one being more intense than the second one. In order to evaluate the area occupied by a puff in the spatio-temporal record provided by the image, a threshold criterion is applied. The mean fluorescence *F* and its standard deviation σ are calculated for the whole (Δ*F*/*F*_*0*_) image. Then all pixels with Δ*F*/*F*_0_ lower than 2*F*_*m*_+10 σ are set equal to 0 and all pixels with fluorescence above that value are set equal to 1. After appliying the threshold, a binary image is obtained for wich the area of the “1 pixels” is calculated. We show in Figure 1B the thresholded image obtained from Figure 1A using this criterion with the “1 pixels” displayed in black and the rest in white. In this way we determine the area occupied by a puff in the spatio-temporal record provided by the image.

**Figure 2:**
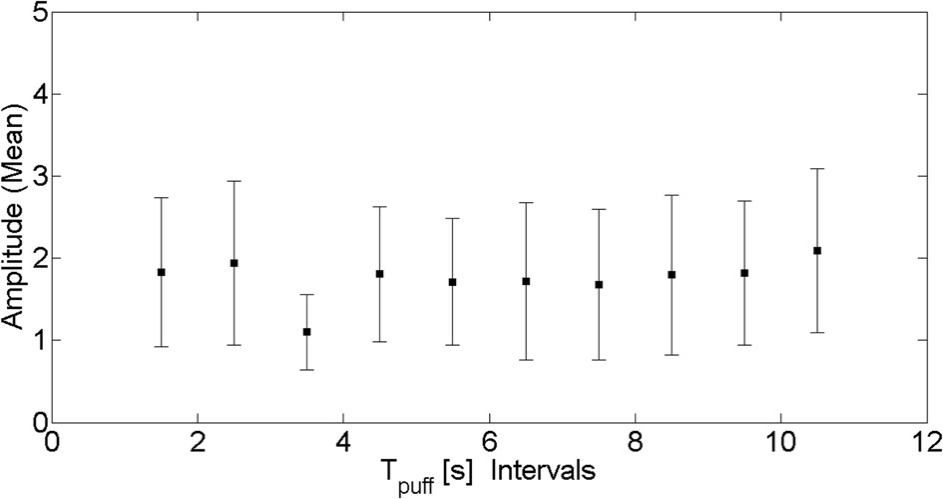
**Puff amplitude as funcion of puff occurrence.** Mean amplitude (symbols) and corresponding standard deviation (vertical lines) of puffs that occurred within the same 1 s interval along the experimental record as a function of the time elapsed since the delivery of the UV pulse.

In order to verify that photobleaching was not affecting our data we grouped together puffs that occurred within the same 1 s-long interval along the experimental record and computed the mean and standard deviation of their amplitude. We show the results in Figure 2 where we can observe that there are no significant changes as time progresses. In this way, we are confident that photobleaching is not affecting the statistics that we are drawing from the experiments.

### 2.4. Data fitting

Some of the data analysed were fitted with different distributions. To evaluate the goodness of those fits, we used the coefficient of determination (ℛ^2^) defined by 1 − *R*_*ss*_/*T*_*ss*_ where *R*_*ss*_ is the residual sum of squares and *T*_*ss*_ is the total sum of squares.

### 2.5. Mathematical models

#### 2.5.1. Intracluster dynamics

In order to obtain the distribution of puff sizes that occur at a given cluster we follow the model introduced in Solovey and Ponce Dawson (2010) using the parameter values that reproduced the observations of Smith and Parker (2009). Briefly, we consider a circular cluster of fixed radius, *R* = 250 nm, over which there are *N* = 18 randomly distributed IP_3_R's. Each IP_3_R has a probability *p* = 5/18 of having IP_3_ bound (i.e., of being *available*). The dynamical phase starts by picking an available channel and assuming it is open. We then open all other available IP_3_R's within a distance *r*_inf_ = 0.250 μm of the open IP_3_R. We iterate the scheme for every new open channel until there are no available IP_3_R's within *r*_inf_ of an open channel that is still closed. We characterize the size of the event by the number of IP_3_R's that became open. Repeating the simulation of our local model for many realizations we produce puff size histograms as shown in other sections. This modeled “size” distribution can be compared with the puff amplitude distribution obtained in our experiments provided that all open IP_3_R contribute equally to the [Ca^2+^] at the cluster. Care must be taken in doing this since this approximation does not always hold (Solovey et al., 2011).

#### 2.5.2. Intercluster dynamics without calcium accumulation

In order to obtain the distribution of event sizes that involve the opening of IP_3_R's in many different clusters inside the cell we use a modified version of the model just described. This modified version assumes that the separation between two channels in a cluster may be neglected when computing their contribution to the free Ca^2+^ concentration at the location of any other cluster and that all the channels of those other clusters sense the same Ca^2+^ concentration at any given time. This assumption relies on the expected difference between lateral cluster size (~200 nm) and inter-cluster distance (~2 μm). Under this assumption, taking into account that inter-channel coupling is due to the Ca^2+^ ions that flow through open IP_3_R's and that Ca^2+^ diffuses with an effective coefficient between neighboring cluster sites we assume that the contribution of a cluster with *N*_*oi*_ open channels located at position *r*_*i*_ to the Ca^2+^ concentration at another position, *r*_*j*_, is proportional to *N*_*oi*_/|*r*_*i*_ − *r*_*j*_|. Then, since at the intra-cluster level we assume that all available IP_3_R's within a distance *r*_inf_ of one open channel become open, at the level of how clusters activate one another we assume that a cluster with *N*_o_ open channels induces the “opening” of all other clusters with available channels that are within a distance *r*_inf_ N_o_ of it. We also assume that, when a cluster is “open” (or active), all its available channels are open (i.e., releasing Ca^2+^). In this scheme we do not add up the Ca^2+^ concentration coming from several open clusters to decide the opening of a new cluster. This simplification is valid as long as the influence of the closest open cluster is more important than that of the more distant ones. The simulations that we show in this paper were done considering a circular total area of radius *R* = 5 μm and *r*_inf_ = 0.250 μm as before. For each realization we selected the number of clusters from a Poisson distribution with mean 25 to guarantee a mean inter-cluster distance of ~2 μm and then determined the number of available channels for each cluster from a Poisson distribution with mean 8. In this way, the fraction of clusters with available channels is similar to the fraction of available to total channels in the case of the intra-cluster simulations.

#### 2.5.3. Intercluster dynamics with calcium accumulation

The previous model of the intercluster dynamics treats every signal as independent of one another. There is evidence, however, that several local signals usually precede a Ca^2+^ (global) wave (Marchant and Parker, 2001). The previous model is then modified to take into account this “coupling” among signals. To that end a larger region (of radius *R* = 20 μm) is considered where a certain number of clusters was randomly distributed with uniform probability over the area. The number of clusters is drawn from a Poisson distribution with mean 400 in order to guarantee a mean inter-cluster distance of ~2 μm and the number of IP_3_-bound IP_3_R's per cluster is drawn from a Poisson distribution of mean 8, as in the previous model. These numbers do not change during the simulation. In order to include signal coupling, we do a time-dependent simulation in which signals are assumed to occur instantaneously and the clusters that participate of it are determined as in the previous intercluster model with some minor modifications. In between signals, the number of active IP_3_-bound IP_3_R's and the Ca^2+^ concentration change with time. Thus, there is an implicit time-scale separation with a fast dynamics during a burst of activity and a slower recovery between such bursts. On the other hand, in this new model we distinguish between active IP_3_-bound and inactive IP_3_-bound IP_3_R's. In particular, we assume that only active IP_3_-bound IP_3_R's can become open during a signal and that all those that participate of an event become inactive immediately after it. Each inactive IP_3_R remains inactive for a certain (random) time. Following (Fraiman et al., 2006) we draw the inhibition time of every inactive IP_3_R from an exponential distribution with mean 2.5 s. At *t* = 0 we assume that all IP_3_-bound IP_3_R's are active and that [Ca^2+^] is equal to [Ca_basal_] = 0.1 μM. It then varies by adding a fixed (spatially homogeneous) amount immediately after the occurrence of a signal per each IP_3_R that opened during the event. [Ca^2+^] subsequently decays exponentially with time towards [Ca_basal_] with rate 200/s. We add a spatially uniform amount to [Ca^2+^] for each open channel under the assumption that Ca^2+^ diffusion occurs fast enough so that [Ca^2+^] becomes approximately uniform in the vicinity of the clusters on a timescale that is much shorter than the mean separation time between events (Fraiman et al., 2006). In order to determine the fixed contribution of each open channel to the total [Ca^2+^] we reason as follows. First, we assume that, while an IP_3_R is open, its Ca^2+^ current is *I* = 0.1pA and that Ca^2+^ diffuses away from it with *D* = 200 μm^2^/s [approximately the free Ca^2+^ diffusion coefficient (Allbritton et al., 1992)]. Thus, when the channel is open it adds a non-uniform contribution Δ[Ca^2+^] to the background [Ca^2+^] that we approximate by the stationary solution of the diffusion equation in the presence of a 2I point source. Namely, Δ[Ca^2+^] ≈ β I μ M μ m^3^/(4π*Dr*) where β is a unit conversion factor and *r* is the distance to the open channel. Given the values of *I* and *D* considered, it is Δ[Ca^2+^] ≈ 0.414 μ M μ m/r. Then, we assume that the total amount of ΔCa^2+^ contained in a cylinder of height *h* and radius *R* which encloses the ER's area of the simulation (π *R*^2^) can be approximated by $h\;\int_{0}^{R}\Delta \;\left[{\text{Ca}}^{2+}\right]\;2\pi dr\text{ = 2}\pi hR0.414\mu \text{M}\mu \text{m}$ while the IP_3_R is open and gets uniformly distributed over the same volume immediately after the channel becomes closed. In this way, we estimate the fixed spatially uniform contribution to [Ca^2+^] of each open channel immediately after it becomes closed as 2 π h R 0.414 μ M μ m/(hπ *R*^2^) = 0.828 μ M μ m/*R* = 0.0414 μ M. Therefore, after a signal with *N*_o_ open channels we add *N*_o_0.0414 μ M to [Ca^2+^]. As mentioned before, this concentration subsequently decays towards its basal value with a rate 200/s that has been estimated as *D*/1 μ m^2^ given that, for each open channel, Δ[Ca^2+^] is close to its basal value at a distance of the order of 1 μm. In this new model the initiation of a signal not only depends on the number of active IP_3_-bound IP_3_R's but also on [Ca^2+^]. In particular, we assume that the probability per unit time that a cluster with *N*_active_ IP_3_R's become open starting a new signal is 0.225s^−1^*N_active_*[Ca^2+^]/Ca_basal_. In this way, this probability takes on the value estimated in (Fraiman et al., 2006) for puff initiation in the presence of basal Ca^2+^. Once the first cluster becomes open, the opening of the other clusters that participate of the signal is decided as before but taking into account the background [Ca^2+^] that is already accumulated. In particular, an open cluster, *k*, with *N*_k_ active channels induces the opening of all other clusters, *i*, with *N*_*i*_>0 active channels that satisfy *N*_*k*_0.414 μ M μ m/*d*_*ik*_ + [Ca^2+^] ≥ 0.414 μ M μ m/*r*_inf_ + [Ca_basal_] where *d*_*ik*_ is the distance between the *i*-th and the *k*-th cluster, [Ca^2+^] is the (accumulated) background Ca^2+^ at the time of the signal and *r*_inf_ = 0.250 μm.

## 3. Intracluster and intercluster percolation using a simple model

In this section we show the results of using the intra and intercluster Models without Ca^2+^ accumulation described in the “Materials and Methods” section to generate distributions of Ca^2+^ signal sizes. As explained in that section, one of these models is used for local signals (puffs) that involve the opening of IP_3_R's within a cluster of ~400 nm sides. The other one models signals that can propagate between clusters that are ~2 μm apart from one another. Both models include a simplified description of the two main dynamical processes that shape the signals: IP_3_ and Ca^2+^ binding, treating each signal as independent of one another. In another section we describe the results obtained when Ca^2+^-mediated signal coupling is included in the intercluster model. Signal types strongly depend on IP_3_ and Ca^2+^ since IP_3_R's need to bind these two species to become open. In the experiments with which we compare the models the cells are loaded with caged IP_3_ that is eventually released by means of a UV flash. In this way, the experimentalist has control over the IP_3_ distribution. Therefore, the models assume that [IP_3_] is fixed. The dynamics of Ca^2+^, on the other hand, is entirely controlled by the cells. Thus, its concentration is very low at the beginning of the experiment and only after a latency a Ca^2+^ ion encounters an IP_3_-bound IP_3_R inducing its opening. Once that happens, the released Ca^2+^ diffuses away from the open channel and can induce the opening of nearby IP_3_R's with IP_3_ bound. The change in [Ca^2+^] due to one open channel is noticeable only in its vicinity. Therefore, the Ca^2+^-mediated inter-channel coupling depends on the distance. This feature is taken into account in both models in a simplified way. In the local model, we assume that each channel opens a nearby IP_3_-bound IP_3_R if it is within a fixed distance, *r*_inf_. In the model of global signals we assume that each cluster that is releasing Ca^2+^ through *N*_o_ open IP_3_R's can induce Ca^2+^ release from a nearby cluster if the latter is within a distance *N*_o_*r*_inf_ of the former.

**Figure 3:**
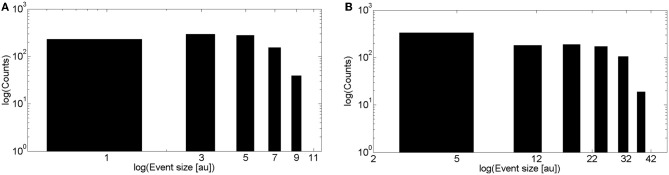
**Event size distributions obtained with simulations of the intra and intercluster models without Ca^2+^ accumulation. (A)** Distribution obtained with 1000 realizations of the intracluster (local) model using the parameters described in the “Materials and Methods” section **(B)** Similar to **(A)**, but for the intercluster model without Ca^2+^ accumulation. See main text for the simulation parameter values.

We show in Figure 3A the puff size distribution obtained with 1000 realizations of the local model using the simulation parameters that allowed us to fit the experimental observations of Smith and Parker (2009). The distribution is relatively flat for small size events and approaches a Poisson distribution for larger events. This behavior is intermediate between two limiting cases: an IP_3_ dominated situation in which all the channels of the cluster that have IP_3_ bound when the event starts participate of the signal. This occurs when IP_3_-bound IP_3_R's are densely packed in the cluster, in which case the size distribution is Poisson. The other limiting case corresponds to events in which IP_3_-bound IP_3_R's are so far away from one another that the opening of one them is not likely to induce the opening of any other available IP_3_R in the cluster. In this case Ca^2+^-binding is the limiting factor for the propagation of the signal. Although there is not a clear phase transition, in Solovey and Ponce Dawson (2010) we referred to the change from the Ca^2+^-binding limited cases to the IP_3_-bound limited cases as percolation since it implies a transition from a situation in which very few channels open to another in which all IP_3_-bound IP_3_R's become open. It is interesting to note that the parameters that best fit the experimental observations are more or less at the border between both limiting cases.

**Figure 4:**
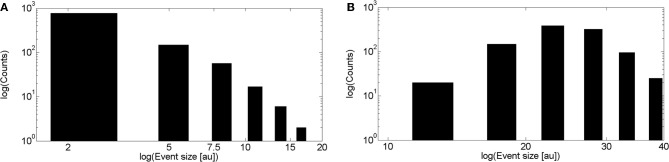
**Transition between Ca^2+^-binding limited and IP_3_-binding limited cases in the event size distribution of the inter-cluster model. (A)** Similar to Figure 3B but using a mean number of IP_3_-bound IP_3_R's equal to 4. **(B)** Similar to **(A)** but using a mean number of IP_3_-bound IP_3_R's equal to 20.

We show in Figure 3B the event size distribution obtained with 1000 realizations of the intercluster coupling model without Ca^2+^ accumulation. The rationale for the parameter values chosen for these simulations is as follows. Given that the simulation covers a ~100 μm^2^ area we chose a mean number of clusters equal to 25 to guarantee a mean inter-cluster separation of around 2 μm. Then, we chose the mean number of IP_3_-bound IP_3_R's in a cluster equal to 8. This is slightly larger than the mean value derived from the observations of Smith and Parker (2009) (6.02 ± 0.48) which correspond to experiments that focused on localized signals. Thus, by choosing a slightly larger value we expect to generate signals that involve the activation of several clusters. Since we assume that all available (i.e., IP_3_-bound) channels of a cluster become open upon activation of the cluster, the mean numbers of IP_3_-bound IP_3_R's and of open channels during a release event, 〈*N*_*o*_〉, in a cluster coincide. Having 〈*N*_*o*_〉=8 gives 〈*N*_*o*_〉 *r*_inf_ =2 μ m for *r*_inf_ = 0.250 μ m as in the intracluster model. For each open cluster, *N*_o_*r*_inf_ is to be compared with the distance to all the other clusters to decide on their activation. Therefore, the parameters are such that *N*_o_*r*_inf_ is, on average, equal to the mean inter-cluster distance. This is similar to the situation encountered for the intracluster dynamics model when using the parameters that gave the best fits to the experiments of (Smith and Parker, 2009). Namely, the mean distance between available channels in the intra-cluster case (~0.12 nm), and the one between clusters, in the inter-cluster case (~2 μm), are of the same order as the numbers they need to be compared with to decide channel opening or cluster activation (*r*_inf_ = 0.250 μm and *N*_o_*r*_inf_, respectively). In fact, we observe that the event size distributions of Figures 3A,B have similar shapes. As in the case of the intracluster dynamics, these parameters of the intercluster model roughly correspond to a mid situation between a Ca^2+^-binding dominated and an IP_3_-binding dominated distribution. This is illustrated in Figure 4 where we display the event size distributions obtained with the same parameters as in Figure 3B but with different values for the mean number of IP_3_-bound IP_3_R's in a cluster. In Figure 4A this value is 4 and intercluster coupling rarely occurs. In Figure 4B the value is 20 and almost all clusters with available IP_3_R's participate of each event.

## 4. Results from experiments

In this section we present the experimental results obtained after analizing puffs from 13 different cells as described in the “Materials and Methods” section. In all the experiments 3 compounds (Fluo4, caged IP_3_, EGTA) were injected in the oocytes. The photorelease of the caged compound was then induced with a UV flash and the resulting fluorescence was measured. All images were obtained in the linescan mode which gives information on the fluorescence along a single line inside the oocyte. A variety of events were elicited in the experiments ranging from small puffs to waves. We only analyzed events that were localized in space and time which limits our experimental distributions. Puff sizes were characterized by their amplitude and (spatio-temporal) area which were computed as described in the “Materials and Methods” section. We present in this section the distributions of these two quantities derived from the experiments.

**Figure 5:**
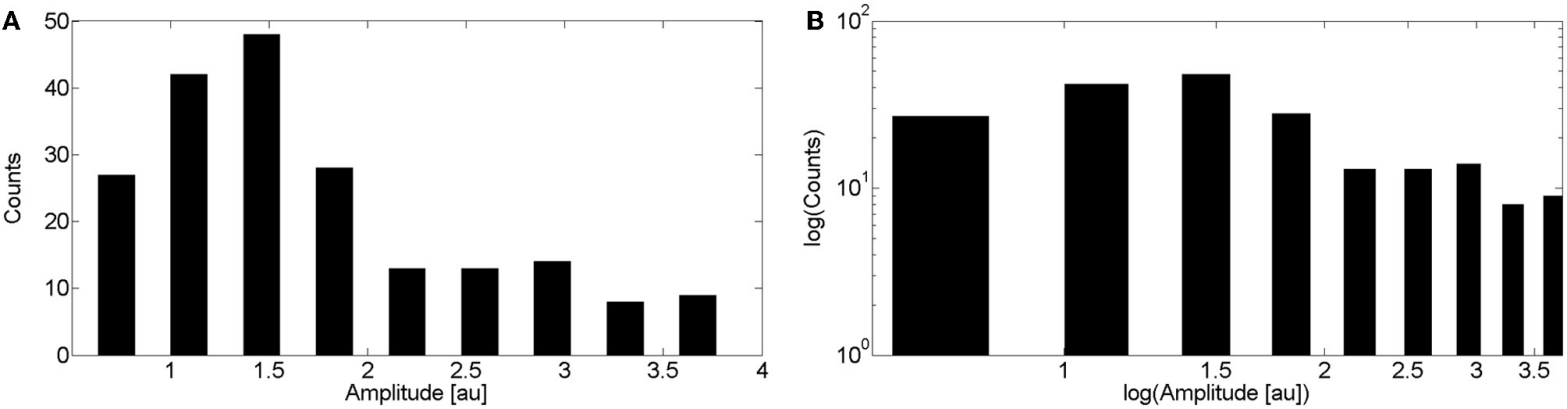
**Distribution of puff amplitudes obtained experimentally. (A)** Distribution obtained by pooling together data from 202 puffs observed in 13 cells. Experiments were performed as explained in the “Materials and Methods” sections. **(B)** Same as **(A)** but on a logarithmic scale.

We show in Figure 5A the puff amplitude distribution computed over 202 puffs obtained from experiments performed in 13 cells. Puff amplitudes varied from 0.54 to 3.95. The distribution shows a single maximum at *A*=1.5. We show in Figure 5B the same distribution but on a logarithmic scale. A power law fitting of these data is not good.

**Figure 6:**
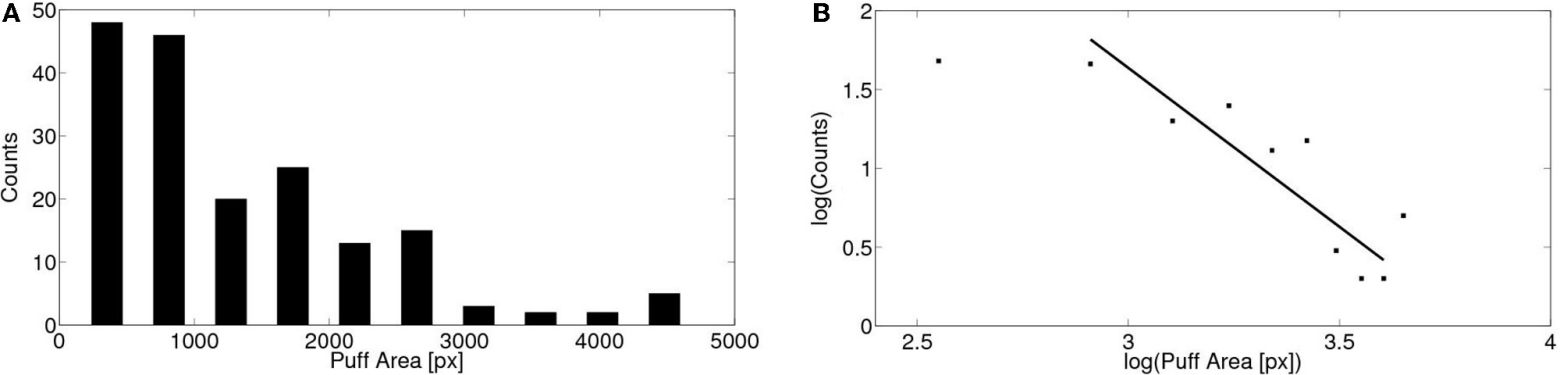
**Distribution of puff areas obtained experimentally. (A)** Similar to Figure 5 but for the area covered by the puffs. Areas were determined as explained in the “Materials and Methods” section pooling data from 179 puffs observed in 12 cells. **(B)** Same as **(A)** but on a logarithmic scale. By fitting a power law relationship to some of the data points we obtain exponents between 1.8 and 2.0 with ℛ^2^ between 0.77 and 0.81 (if we include data between 800 and 4500 px or between 800 and 4000 px, respectively).

We show in Figure 6A the corresponding distribution of puff areas (spatio-temporal spread). Reliable area values could only be obtained for 179 puffs observed in 12 oocytes. Puff areas were estimated as explained in the “Materials and Methods” section. The same distribution is shown on a logarithmic scale in Figure 6B. In this case, the distribution can be approximated by a power law of exponent between 1.7 and 2.0 depending on the range of areas included in the fitting. The goodness of the fit was estimated by the coefficient of determination ℛ^2^, which varied from 0.77 to 0.81 depending on the range of areas included. We compared this value with the ones obtained by fitting the data with other distributions. The goodness was worse in all cases with the exception of the log-normal distribution where we obtained ℛ^2^ = 0.90. It is always difficult to distinguish power law and log-normal distributions (Clauset et al., 2009) and in this case it is even more difficult given the small range of events spanned by the distribution. In either case, a key feature of both types of distributions is the existence of a long tail. As discussed later, in the case of calcium signals, this is a relevant property for its possible physiological implications.

## 5. Calcium accumulation and IP_3_R inhibition can lead to a size distribution that can be fitted by a power law

In the previous section experimental signal sizes of mainly local signals were characterized by two quantities: their amplitude and their spatio-temporal spread. These quantities not only depend on the number of IP_3_R's that open during the signal but also on the times of the openings and their spatial distribution (Solovey et al., 2011; Diambra and Marchant, 2011). In any case, they can be compared with the distributions of channels that participate of the events obtained from simulations of the different models. In particular, neither the models nor the experimental puff amplitude distributions can be well described by a power law. The experimental distribution of puff areas, however, could be fitted by a log-normal distribution or a power law, albeit over a relatively short range of lengthscales. The limited range of lengthscales could be attributed to the fact that we have mainly analyzed local signals. Had we included events that involved the recruitment of IP_3_R's from several clusters we might have obtained a power law over a larger range. Signal areas can readily be associated with the size of “avalanches” in typical examples of systems that display SOC. The ability to fit the distribution of Figure 6B with a power law could be an indication that IP_3_R-mediated Ca^2+^ signaling in oocytes displays SOC as Ca^2+^ waves in astrocytes (Jung et al., 1998) and in cardiac myocytes Nivala et al. (2012). This power law behavior, however, is not captured by the model distributions of Figure 3. The (spatio-temporal) area covered by a signal is strongly dependent on Ca^2+^-mediated channel and cluster coupling. There is experimental evidence, on the other hand, that several local signals usually precede a Ca^2+^ (global) wave when cells are continuously subjected to IP_3_ release (Marchant and Parker, 2001). Although cells have very efficient mechanisms to return Ca^2+^ back to basal once a signal has finished this does not occur immediately. Thus, there can be a slow Ca^2+^ accumulation in between signals which has in fact been observed (Marchant and Parker, 2001). This remaining Ca^2+^ effectively couples local signals, an effect that is not taken into account in the intra and intercluster models probed in section 3. Furthermore, IP_3_R's become inactive after having opened. The typical inhibition time has been estimated to be of the order of 2s (Fraiman et al., 2006). This introduces an additional inter-signal coupling. In order to take these two effects into account we modified the intercluster model simulated in section 3. As explained in the “Materials and Methods” section, in order to mimic signal coupling, we do a time-dependent simulation in which we distinguish between active and inactive IP_3_-bound IP_3_R's and where signals are assumed to occur instantaneously. In between signals, the number of active IP_3_-bound IP_3_R's (the only ones that can become open during a signal) and the Ca^2+^ concentration change with time. Thus, the model has an implicit time-scale separation with a fast dynamics during a burst of activity and a slower recovery between such bursts. IP_3_R's become inactive after having opened and go back to being active with a timescale of the order of 2 s. [Ca^2+^], on the other hand, increases after a signal depending on how many channels were open and then decays back to its basal value with a rate of the order of 200s^−1^. For more details on this new model we refer the reader to the “Materials and Methods” section.

**Figure 7:**
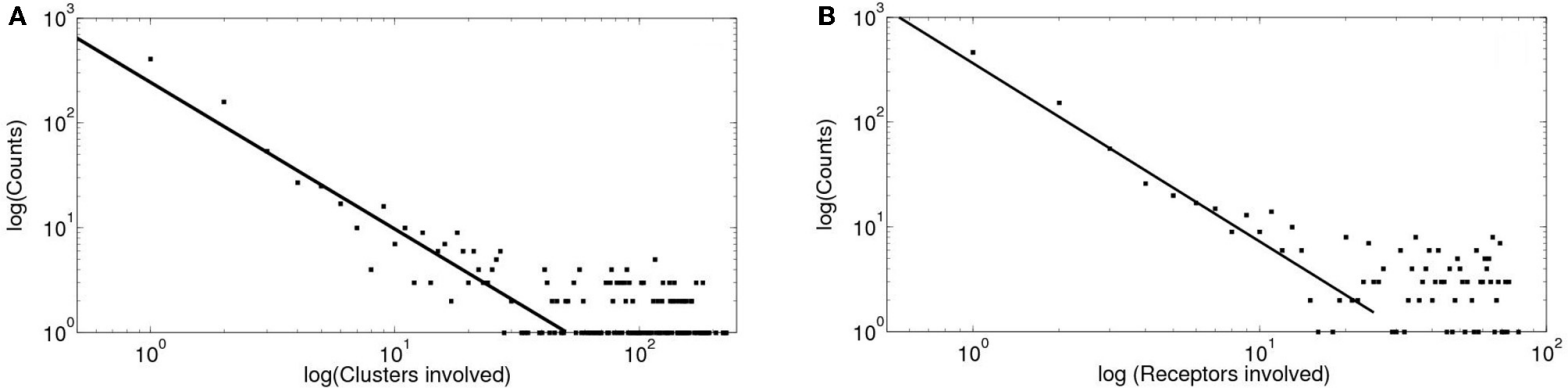
**Distributions of clusters and of receptors involved obtained with simulations of intercluster dynamics with calcium accumulation. (A)** Distribution of the number of clusters involved in each signal displayed on a logarithmic scale. By fitting a power law relationship to some of the data points we obtain exponents between 1 and 1.4 with ℛ^2^ between 0.72 and 0.85 (if we include data between 30 and 80 clusters involved, respectively). **(B)** Distribution of the number of IP_3_R's involved on a logarithmic scale. By fitting a power law relationship to some of the data points we obtain exponents between 1 and 1.7 with ℛ^2^ between 0.76 and 0.92 (if we include data between 20 and 80 IP_3_R's involved, respectively).

We show in Figure 7 the results obtained with the intercluster model that takes signal coupling into account. In this case we decided to characterize the signal size by both the number of clusters and the number of IP_3_R's (receptors) that participated of each event. We show the distributions of these two quantities in Figures 7A,B on a logarithmic scale. For this model these two distributions can be approximated by a power law over a relative large range of sizes. Furthermore, the exponent for the distribution of the number of channels is within the range of the one determined from experiments for the puff area distribution. We also fitted these distributions according to the methods described in Clauset et al. (2009) and obtained similar results. The power law approximation fails at large sizes probably due to a finite size effect. Both distributions were not well fitted by other distributions such as log-normal or log-logistic.

## 6. Mean cytosolic calcium provides a global coupling mechanism that alters the signal size distribution

**Figure 8:**
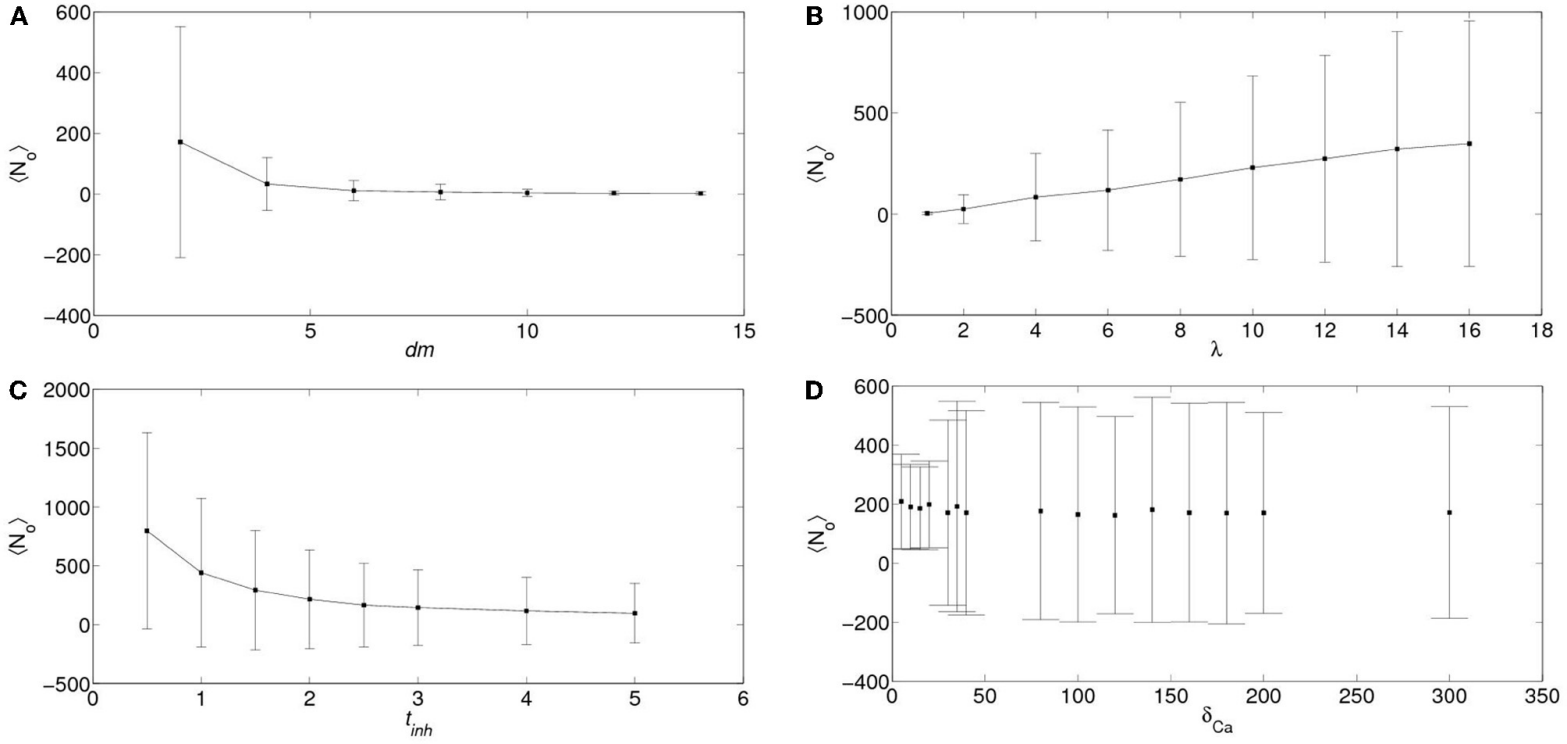
**Dependence of the number of IP_3_R's that participate of a signal on various parameters.** Mean (symbols) and standard deviations (vertical lines around mean) of the number of IP_3_R's that participate of a signal, *N*_o_, as a function of the inter-cluster distance, *dm* (**A**), of the mean number of IP_3_-bound IP_3_R's per cluster, λ (**B**), of the mean IP_3_R inhibition time, *t*_inh_ (**C**), and of the cytosolic Ca^2+^ clearing rate, δ_Ca_ (**D**). In all the subfigures, the parameters that are not varied are fixed at the values used in section 5.

**Figure 9:**
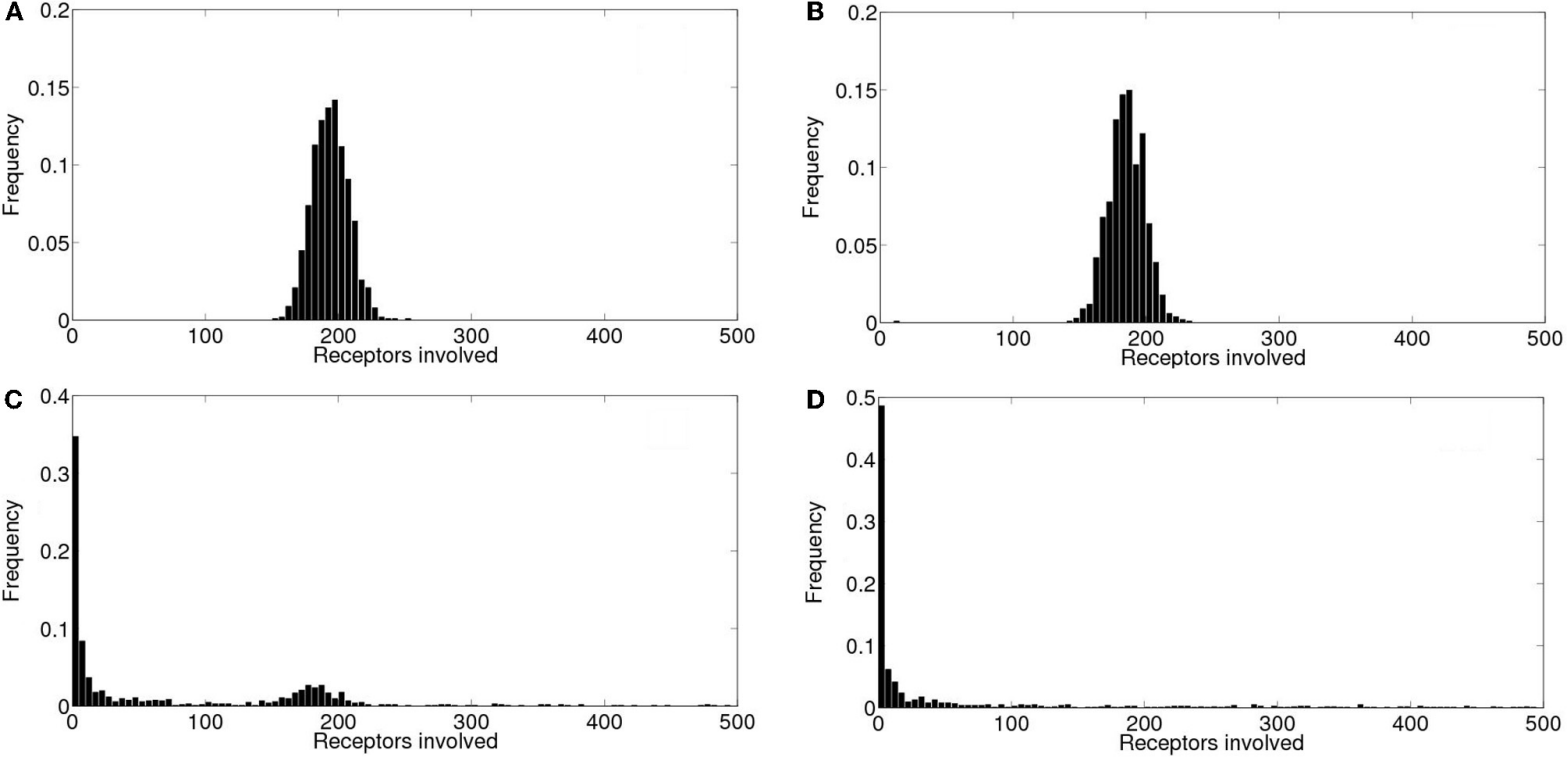
**Event size distributions obtained for different values of the cytosolic Ca^2+^ clearing rate.** Distribution of the number of IP_3_R's that participate of the signals when all the parameters are kept at the values used in section 5 except for the cytosolic Ca^2+^ clearing rate for which we used: (**A**) δ_Ca_ = 20/s, (**B**) δ_Ca_ = 25/s, (**C**) δ_Ca_ = 30/s, (**D**) δ_Ca_ = 200/s.

In order to analyze the robustness of the power law behavior encountered in the previous section we simulated the model for other values of its various parameters. More specifically, we studied how the distributions of the number of event-participating clusters and IP_3_R's changed with the mean inter-cluster distance, *dm*, the mean time an IP_3_R remains inhibited, *t*_inh_, the rate at which cytosolic Ca^2+^ decays to its basal value, δ_Ca_ and with the radius of influence of an open channel, *r*_inf_ which were *dm*= 2 μm, *t*_inh_ = 2.5s, δ_Ca_ = 200/s and *r*_inf_ = 0.250 μm in all the simulations of the previous section. We found that in almost all cases the distributions could be fitted by a power law (with exponents between 1 and 1.8) but over a range of event sizes that could be different depending on the parameter values. This is reasonable since the largest event size is strongly dependent on some of these parameters. For example, for large values of the intercluster distance, *dm*, or of the inhibitory time, *t*_inh_ and for small values of the mean number of IP_3_-bound IP_3_R's per cluster, λ, the largest event size decreased significantly. Varying *r*_inf_ between 0.01 μm and 0.1 μm did not produce any noticeable changes. While varying the cytosolic Ca^2+^ clearing rate, δ_Ca_, between 50/s and 100/s did not change the power law behavior of the signal size distribution, using smaller values (we probed 20/s ≤δ_Ca_≤ 30/s) produced a striking change. Namely, the event size distribution ceased to be long-tailed and looked more like Gaussian. We illustrate the results obtained in Figures 8 and 9. We show in Figure 8 the mean and standard deviation of the number of IP_3_R's that participate of the signals, *N*_o_, as a function of a varying parameter when all the others are kept fixed at the values used in the previous section. We can observe in this Figure the trends we have just described with respect to how the mean number of participating (open) IP_3_R's, *N*_o_, varies with *dm*, λ and *t*_inh_. We also observe that, when varying any of these 3 parameters the standard deviation of *N*_o_ decreases with decreasing *N*_o_. We have also plotted *N*_o_ and σ_No_ normalized to the initial number of active IP_3_R's in the simulation obtaining a similar behavior (data not shown). The behavior encountered for small vallues of δ_Ca_ is quite different. Namely, the standard deviation is much smaller than for all the other sets of parameter values explored. Furthermore, the deviation, σ_No_, decreases with increasing 〈*N*_*o*_ 〉. This is the consequence of the distribution no longer being long-tailed. This can be observed in Figure 9 where we have plotted the distribution of the number of IP_3_R's that participate of the signals when all the parameters are kept at the values used in the previous section except for the cytosolic Ca^2+^ clearing rate for which we used δ_Ca_ = 20/s (A), δ_Ca_ = 25/s (B), δ_Ca_ = 30/s (C) and δ_Ca_ = 200/s (D). There we observe that for δ_Ca_ large enough the distribution is well approximated by a power law. As δ_Ca_ decreases, this power law behavior still holds for small enough *N*_o_ but a new “Gaussian-like component” seems to build in the large *N*_o_ region. Eventually, when δ_Ca_ is small enough, the power law region disappears and we obtain a Gaussian distribution.

**Figure 10:**
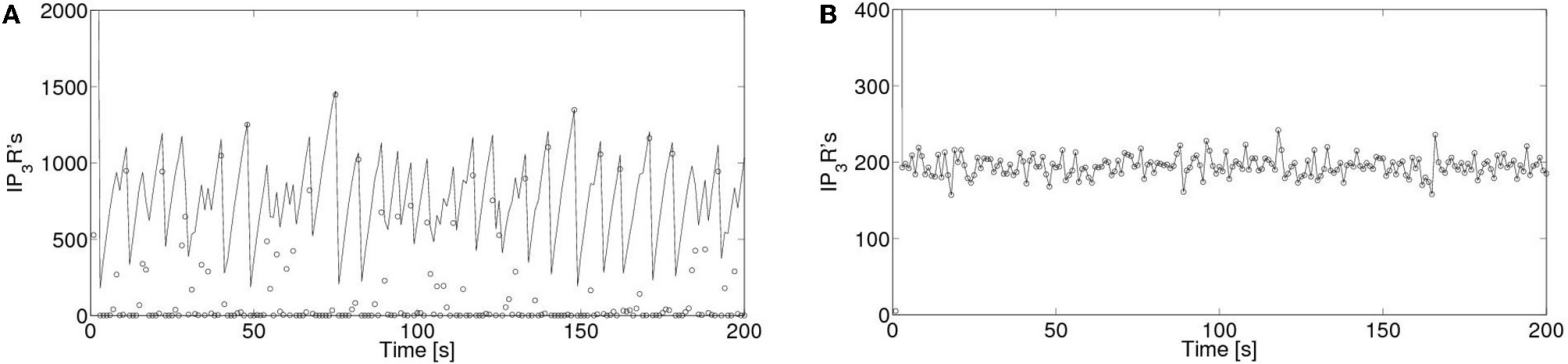
**Number of active IP_3_R's and of IP_3_R's that are participating of a signal as a function of time.** Number of active IP_3_R's, *N*_act_ (with solid lines) and of open IP_3_R's, *N*_o_ (with open circles) as a function of time for **(A)** δ_Ca_ = 200/s and for **(B)** δ_Ca_ = 20/s.

In order to interpret this result, we compared the number of receptors that participate of each event, *N*_o_ and the number of IP_3_R-bound IP_3_R's that are active, *N*_act_ at any given time. We show a plot of these two numbers as a function of time for δ_Ca_ = 200/s Figure 10A and for δ_Ca_=20/s in Figure 10B. We observe that while, in the former *N*_o_ < N_act_ for most events, for δ_Ca_=20/s all active receptors participate of each event (*N*_o_ = N_act_). In this way, the distribution of event sizes coincides with that of active channels which, after a short transient, has a well defined mean value with a relatively small deviation around it. The event size distribution then looks Gaussian.

## 7. Discussion and conclusions

Intracellular Ca^2+^ signals are ubiquitous across cell types. Among them, those that involve Ca^2+^ release from the ER through IP_3_ receptors (which are Ca^2+^ channels) play a relevant role. Apparently, activatable IP_3_R's are distributed in clusters on the surface of the ER. This uneven distribution together with the kinetic properties of the IP_3_R according to which the channel needs to bind Ca^2+^ and IP_3_ to become open give rise to a variety of signals that go from very localized ones (puffs) to global waves that propagate throughout the cell. Ca^2+^-mediated channel coupling due to CICR thus plays a key role to determine the spatial size over which the signal spreads. The sequence of channel openings during a signal is then similar to the processes involved in percolation. In particular, we used these ideas in Solovey and Ponce Dawson (2010) to analyze a local signal generation model (i.e., limited to one cluster of IP_3_R's). In Solovey and Ponce Dawson (2010) we called percolation the transition between a situation for which most signals involved the opening of all IP_3_-bound IP_3_R's of the cluster (percolating states) to a situation for which very few IP_3_R's of the cluster opened (non-percolating states). By fitting the model to the experimental results of Smith and Parker (2009) (which were obtained in intact mammalian cells of the human neuroblastoma SY5Y cell line) we determined in Solovey and Ponce Dawson (2010) that the experiments seemed to be at a transition point between percolating and non-percolating states. In the present paper we obtained a similar result when looking at the distribution of puff amplitudes determined from experiments performed in *Xenopus Laevis* oocytes. The experimental distribution of puff (spatio-temporal) areas in this cell type, on the other hand, was slightly different and could be fitted by a power law (goodness of fit) or a log-normal distribution (goodnes of fit). It is always difficult to distinguish power law and log-normal distributions (Clauset et al., 2009). In the present case, it is especially difficult given the small range of event sizes spanned by the experiments which correspond mainly to local signals. However, since puff area is a better reporter than puff amplitude of Ca^2+^-mediated channel coupling when channels are relatively apart from one another we expect a similar type of behavior to be encountered if signals that involve channel recruitment from several clusters are also included in the statistics. In this way, the range of sizes would be larger and a stronger indication on the type of distribution could be drawn. In fact, the power law distributions typical of SOC have been observed for Ca^2+^ waves in astrocytes (Jung et al., 1998) and in cardiac myocytes (Nivala et al., 2012).

In order to investigate the size distribution of signals that involve the opening of IP_3_R's in many clusters we extended the model of Solovey and Ponce Dawson (2010) to include inter-cluster coupling. The first modification that we tried treated each signal independently of one another. It led to a similar distribution to that of local (intracluster) signals which does not follow a power law. This first extension of the model, however, is not suitable for situations in which cells are constantly challenged with a given stimulus as done in Marchant and Parker (2001). Assuming that the signals act independently of one another is not very realistic in such a case. It has been reported that several local signals usually precede Ca^2+^ waves, particularly when the cells are subjected to a constant release of IP_3_ as done in Marchant and Parker (2001). This is an indication that, upon a continuous stimulation, signals can affect one another, especially when they involve the opening of many channels in various different clusters. In order to take this inter-signal coupling into account we further extended the intercluster model including both the dynamics of the global cytosolic [Ca^2+^] and of the activation and inactivation of IP_3_-bound IP_3_R's upon channel opening. Using realistic parameter values we obtained distributions for the number of clusters and for the number of channels which participate of the signals that can be fitted by a power law with exponents that are within the values obtained for the experimental spatial puff size distribution. We probed the robustness of this behavior by changing the values of various parameters of the model (the mean inter-cluster distance, *dm*, the mean time an IP_3_R remains inhibited, *t*_inh_, the rate at which cytosolic Ca^2+^ decays to its basal value, δ_Ca_ and the radius of influence of an open channel, *r*_inf_). In particular, we determined that the distributions could still be fitted by a power law if we changed any of these parameters around the values used in the original simulations. This robust behavior could lead to the interpretation that our model and thus, the Ca^2+^ signals it describes, display SOC. Such a conclusion would not be surprising given that the system under study has the main “ingredients” with which a SOC state can be reached (Dickman et al., 2000; Vespignani et al., 2000). Namely, it is a system with a phase transition onto an absorbing (inactive) state (the state with all channels closed) that is driven slowly by infinitesimal additions of the quantity that brings the system close to the transition to the active state (in our case there are two factors that affect this transition: global cytosolic [Ca^2+^] and the number of IP_3_-bound IP_3_R's that are active at any given time) and which is allowed to relax upon activation by decreasing this quantity (in our case the number of active IP_3_-IP_3_R's which drops immediately after a signal is elicited). In any case, as already mentioned, it is always difficult to establish with certainty that a given distribution follows a power law or not. In the case of the experimental data that we analyzed the log-normal distribution gave a better fit. However, this could be due to the limited amount of data that we have. It is in fact quite difficult to distinguish a power law from a log-normal distribution (Clauset et al., 2009). It has been shown, for example, that multiplicative processes which naturally lead to log-normal distributions give rise to power law distributions if there is a bounded minimum that acts as a lower barrier to the multiplicative model (Mitzenmacher, 2004). In the case of Ca^2+^ signals, there is a lower limit to event sizes (which corresponds to the size of a single channel opening) and one could argue that this could favor a power law over a log-normal distribution. From the point of view of the mechanisms that could lead to one or the other situation, our modeling results seem to favor the power law distribution as well. However, given that our model is only an approximation to the real problem, we cannot tell for sure that IP_3_-mediated Ca^2+^ signals are an example of SOC. We are certain, however, that the signal size distribution has a long tail and this could have physiological implications as we discuss later.

The event size distribution was not long-tailed for all the parameter values that we probed with our model. In particular, when the cytosolic Ca^2+^ clearing time, δ_Ca_, was decreased by an order of magnitude we obtained a Gaussian-like distribution around a mean for both the number of IP_3_R's (Figure 9) and of clusters (data not shown) that participate of each signal. We analyzed how the mean and standard deviation of the number of participating IP_3_R's varied with each of the above mentioned parameters. We found that, in most cases probed, the deviation increased with the mean. The exception was the situation encountered for small δ_Ca_ for which we obtained that the deviation, σ_No_, increased slightly while the mean, 〈*N*_*o*_〉, decreased when δ_Ca_ increased up to a point at which a large increase in σ_No_ was observed (Figure 8). This signals a transition from a situation with a Gaussian-like distribution to a situation for which the distribution was long-tailed and could be fitted by a power-law (at least over some range of signal sizes). In order to understand why the different behaviors are obtained we compared the number of active IP_3_R's, *N*_act_, and that of IP_3_R's that participate of a signal, *N*_o_, at any given time. We observed that, while for those cases in which the distribution is long-tailed, *N*_o_ was less than *N*_act_ for most events, the number of participating IP_3_R's was equal to that of active ones for almost all events in the case of the Gaussian-like distribution. In particular, we achieve this situation decreasing δ_Ca_ because, in that way, the cytosolic Ca^2+^ concentration remains high enough so that no matter how small the addition of the Ca^2+^ released by an open IP_3_R is, all IP_3_R's become open as soon as they become active. Thus, in this situation inter-channel coupling plays almost no role. As discussed in Cui et al. (2009), for a model of coupled excitable units that are spatially distributed and that, when “firing” (become open) can induce the “firing” of their active neighbors and then become inactive for a certain refractory time, random openings turn into a random distribution of active units in space. In this way, after a signal that involves the opening of almost all available units, it is very rare that a newly open unit has another active unit in its neighborhood to induce its opening and the signal will fail to propagate. Thus, most likely small signals will follow until a large enough number of IP_3_R's become active again. The random and spatially patchy distribution of active excitable units is a basic ingredient for the existence of a long-tailed distribution of size events as the one observed in our model and in Nivala et al. (2012). This is also key for the large variability of the time interval in between large “spikes” as has been observed in Marchant and Parker (2001); Skupin et al. (2008). Forcing such a system with an external periodic stimulus (like what happens in cardiac myocytes) can lead to a period doubling instability Cui et al. (2009) which can explain Ca^2+^ alternans (Jia et al., 2012). When all units are globally coupled (or, rather, they are continously subjected to an environment that make them “fire” as soon as they become active) the random sparse spatial distribution of excitability disappears, the distribution of event sizes ceases to be long-tailed as observed in our model and the period-doubling instability described before disappears. We have observed that the cytosolic Ca^2+^ concentration provides a very effective way to switch from a sparsely connected system in which the signal size distribution is long-tailed and may be approximated by a power law to a globally coupled system with a Gaussian-like event size distribution. This last behavior resembles what may observed in the experiments of Marchant and Parker (2001): when a large concentration of IP_3_ is continuously released, basal Ca^2+^ accumulates and the variability of interspike intervals becomes negligible. Situations with a more efficient coupling mechanism but that had to rely on signal propagation to produce a large event (e.g., setting *dm* = *r*_inf_ but keeping λ=1) failed to give a purely Gaussian distribution giving instead a mixture of a power law for small events and a Gaussian for large ones. Our experiments and those of Marchant and Parker (2001) were performed in immature oocytes. It is known that, with maturation, the ER is reconfigured and the IP_3_R spatial distribution changes which, in turn, leads to Ca^2+^ signals that tend to spread over a larger spatial region (Machaca, 2004, 2007). It has been argued that an increase of IP_3_R sensitivity could underlie this behavior of the signals (Ullah et al., 2007). Most likely it is a combination of causes which final effect is to couple IP_3_R's more efficiently preventing the random patchiness of excitability observed in immature oocytes to occur. In this way the mature egg becomes ready to be fertilized and a Ca^2+^ wave can propagate without failure (Whitaker, 2006).

Although further studies are necessary to verify our findings, the results obtained in this paper show under what circumstances intracellular Ca^2+^ signals can have a long-tailed (SOC-like) size distribution and in which ways this can be changed.

### Conflict of interest statement

The authors declare that the research was conducted in the absence of any commercial or financial relationships that could be construed as a potential conflict of interest.
